# Reimbursement in the age of generalist radiology artificial intelligence

**DOI:** 10.1038/s41746-024-01352-w

**Published:** 2024-12-02

**Authors:** Siddhant Dogra, Ezequiel “Zeke” Silva, Pranav Rajpurkar

**Affiliations:** 1 Department of Radiology, New York University Langone Health, New York, NY USA; 2 South Texas Radiology, San Antonio, TX USA; 3 University of Texas Health, Long School of Medicine, San Antonio, TX USA; 4 Department of Biomedical Informatics, Harvard Medical School, Boston, MA USA

**Keywords:** Medical imaging, Health policy

## Abstract

We argue that generalist radiology artificial intelligence (GRAI) challenges current healthcare reimbursement frameworks. Unlike narrow AI tools, GRAI’s multi-task capabilities render existing pathways inadequate. This perspective examines key questions surrounding GRAI reimbursement, including issues of coding, valuation, and coverage policies. We aim to catalyze dialogue among stakeholders about how reimbursement might evolve to accommodate GRAI, potentially influencing AI reimbursement strategies in radiology and beyond.

## Introduction

As the number of radiology artificial intelligence (AI) / machine learning (ML) devices approved by the US Food and Drug Administration (FDA) continues to rise, there has been an increased emphasis on integrating these tools into clinical practice^1^. While larger health systems and radiology groups will be able to develop homegrown AI models or buy commercial tools, true widespread integration and implementation will require development of a comprehensive AI payment system. Although a handful of AI/ML devices have already been approved for reimbursement, these devices and current radiology solutions in general can be classified as narrow AI solutions that are designed to focus on specific, predefined tasks, such as pulmonary embolism triage^2^. More recently, there has been increased excitement regarding advances in foundation model curation allowing for development of generalist radiology AI (GRAI), which fundamentally differs from narrow AI and will likely necessitate different reimbursement strategies^3^.

Definitionally, GRAI extends upon the recently introduced concept of generalist medical AI, which is based on three fundamental capabilities: it will be able to adapt to new tasks, described with natural language, without being retrained; it will be able to use multiple data modalities as inputs as well as outputs; and will be able to explain the reasoning behind outputs using medical reasoning and terminology^4^. Of course, GRAI needs features specific to radiology tasks, for example the ability to produce comprehensive reports tailored to indications adjusted for clinical context. Consequently, GRAI would offer more comprehensive, multi-task tools better equipped to help radiologists and other clinicians. The most advanced radiology generalist models have already been shown to demonstrate these capabilities and outperform specialized models. For example, MedVersa is a generalist tool that performs well in multiple radiology tasks such as segmentation, detection, and chest radiograph report generation^5^.

Given the research successes of and interest in GRAI development, we should expect commercial GRAI tools to emerge in the coming years. GRAI reimbursement will presumably need to be considered differently from reimbursement for narrow AI, given intrinsic differences in model development and applications. Consequently, we should begin thinking about how reimbursement may look in the age of GRAI. In this perspective, we briefly review how reimbursement is handled both generally within medicine as well as for AI and posit how GRAI may fit into this scheme.

## Basics of reimbursement

**Fig. 1:**
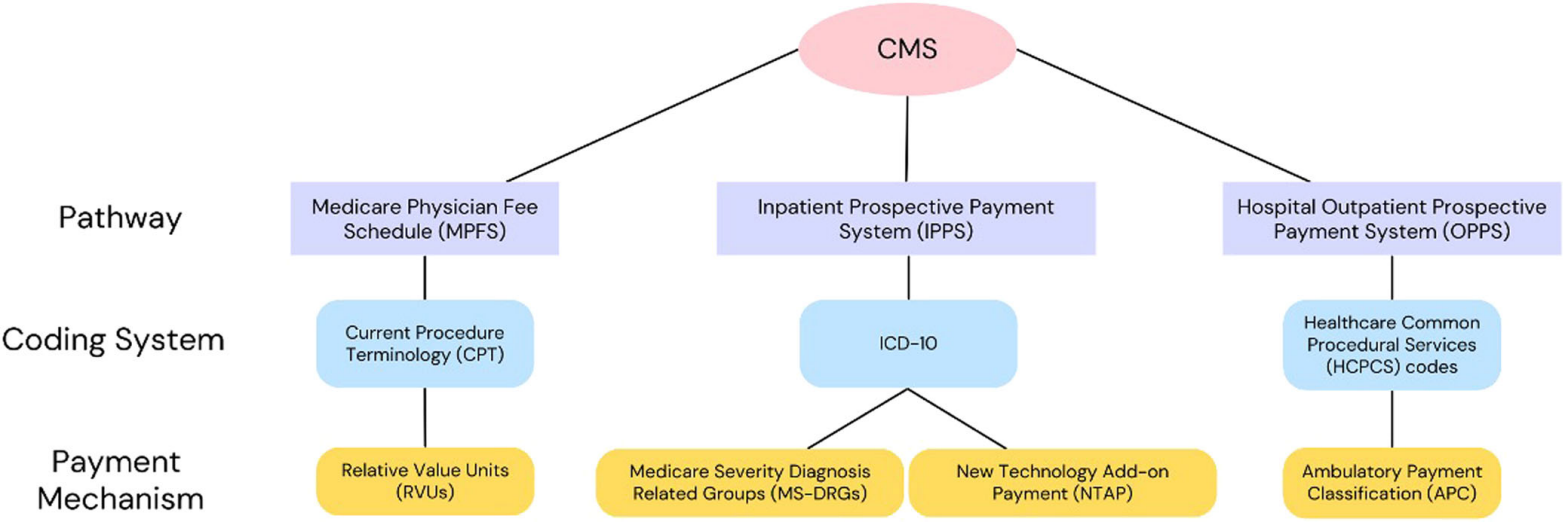
Current reimbursement pathways. Flowchart of reimbursement pathways used by the Centers for Medicare and Medicaid (CMS). ICD International Classification of Diseases.

The Centers for Medicare and Medicaid (CMS) is the single largest payor in the United States, reimbursing physicians, hospitals, ambulatory surgery centers, outpatient physician offices and others for services provided to Medicare beneficiaries (Fig. 1). Given the volume of physician services which are paid through the Medicare Physician Fee Schedule (MPFS), which covers physician office payments, the MPFS will be an important pathway for AI payment to physicians and will be emphasized in this article. Hospital inpatient and outpatient services to Medicare beneficiaries are paid through the Inpatient Prospective Payment System (IPPS) and Hospital Outpatient Prospective Payment System (OPPS), respectively.

CMS maintains a list of Current Procedural Terminology (CPT) codes, a subset of the larger Healthcare Common Procedural Coding System (HCPCS), to identify services and procedures provided to Medicare beneficiaries. These codes are used within the MPFS and OPPS pathways. The American Medical Association (AMA) maintains CPT codes through its CPT Editorial Panel (CPT EP). The two most common types of CPT codes are Category I and Category III. Category I applies to services which satisfy the following criteria: (1) approved by the FDA; (2) strength of evidence; and (3) widely performed. Codes which do not satisfy all three of these criteria may achieve Category III status as emerging technologies^6^. Category II codes are used for performance management. In comparison, inpatient service payments are initially classified via ICD-10 (International Classification of Diseases) codes, and then assigned to Medicare Severity Diagnosis Related Groups (MS-DRGs) to determine payment. Novel inpatient healthcare services may also merit a New Technology Add-on Payment (NTAP), a temporary payment superimposed on the MS-DRG payment for up to three years^7^. Outpatient payments via the OPPS pathways relies on CPT and HCPCS coding, using organized groupings called Ambulatory Payment Classifications (APCs) as the payment mechanism.

Payment within the MPFS is based on the Resource Based Relative Value Scale (RBRVS). The RBRVS is maintained by the AMA Multi-Specialty RVS Update Committee (RUC) which makes relative value recommendations to CMS. CMS has the final determination on acceptance of the relativity recommendations of the RUC, but historically CMS has accepted approximately 90% of the RUC’s recommendations^8^. The relative value of a service within the RBRVS is based on Relative Value Units (RVUs). The Total RVU for a given service includes three components: Work, Practice Expense, and Malpractice, each with separate RVU determinations and assignments. The practice expense RVU includes two types of inputs: Direct and Indirect. Direct inputs are CPT code specific and include clinical staff, disposable supplies, and equipment. Indirect inputs are more general, such as utilities or reception staff and are informed by the AMA administered Physician Practice Information Survey (PPIS). The PPIS was administered and applied in the late 2000s; however, an update to the PPIS is ongoing. The broader practice expense methodology employed by CMS is complex with multiple adjustments and scaling factors applied to final payment amount determination^9^.

## Existing approaches for AI reimbursement

Payment for AI services across all specialties is evolving. The FDA defines Software as a Medical Device (SaMD) as “software intended to be used for one or more medical purposes that perform these services without being part of a hardware medical device.”^10^ ML algorithms can be classified as SaMD, although only two ML algorithms have thus far received a category I CPT code: 92229 (Imaging of retina for detection or monitoring of disease; with point-of-care automated analysis with diagnostic report; unilateral or bilateral); and 75580 (Coronary fractional flow reserve (FFR) derived from augmentative software analysis). However, five other SaMD algorithms have received Category III status: +0764 T and 0765 T (Assistive algorithmic electrocardiogram risk-based assessment for cardiac dysfunction); 0794 T (Patient-specific, assistive, rules-based algorithm for ranking pharmaco-oncologic treatment options); and 0731 T Augmentative AI-based facial phenotype analysis with report; +0857 T Opto-acoustic imaging, breast, augmentative analysis and report; 0740 T Remote autonomous algorithm-based recommendation system for insulin) (Table 1).

**Table 1:** SaMD Machine Learning Algorithms

CPT Code	Algorithm Name	CPT Code Category
92229	Imaging of retina for detection or monitoring of disease; with point-of-care automated analysis with diagnostic report; unilateral or bilateral	I
75580	Coronary fractional flow reserve (FFR) derived from augmentative software analysis	I
+0764 T, 0765 T	Assistive algorithmic electrocardiogram risk-based assessment for cardiac dysfunction; includes analysis, with report (*0765* *T has patient-initiated transmission*)	III
0794 T	Patient-specific, assistive, rules-based algorithm for ranking pharmaco-oncologic treatment options; includes report	III
0731 T	Augmentative AI-based facial phenotype analysis with report	III
+0857 T	Opto-acoustic imaging, breast; with augmentative analysis and report	III
0740 T	Remote autonomous algorithm-based recommendation system for insulin therapy, including data collection, monitoring, and adjustment of insulin dosage	III

Machine learning algorithms with Current Procedural Terminology (CPT) codes that are classified as Software as a Medical Device (SaMD).

The AMA CPT Editorial Panel created an AI taxonomy for medical services and procedures to enable consistency in the nomenclature for this growing class of services. Appendix S describes these services in one of three categories: (1) Assistive; (2) Augmentative; and (3) Autonomous^11^. It is expected that future code change applications for artificial/augmented intelligence applications shall consult this taxonomy during the submission process. As AI tools grow more sophisticated, they will presumably progress further along these categories^12^.

The first AI CPT code was created for LumineticsCore, formerly IDx-DR, for detection and diagnosis of diabetic retinopathy from retinal images. Examination of the LumineticsCore CPT code helps identify challenges associated with CPT code development for AI^13^. The major components of the RVU for CPT codes are physician work and practice expenses. As an autonomous tool, no physician work is required for LumineticsCore, so that component is zero. Other tools that assist or augment physician work may just be considered as part of expected work within an existing code, even though some algorithms may increase physician time as what was experienced with mammographic computer-aided detection^14^. Practice expense is divided into direct (can be tied to a single patient encounter like technologist time or equipment) or indirect (not for a specific encounter such as rent). Unfortunately, software costs like those required for LumineticsCore are difficult to characterize in this regard^15^, and CMS has acknowledged that their practice expense methodology is not well-suited for AI applications.

On the inpatient side, the NTAP was explicitly designed as a way to incentivize hospitals to adopt innovative technologies that may otherwise increase costs. The first approved AI NTAP application request was for a triage and notification system for detection of large vessel occlusions created by Viz.ai^13^. However, other NTAP requests such as for triage and notification of suspected pulmonary embolism as well as for calculation of ASPECTS score from CT head studies performed for suspected stroke have been rejected, as these applications were thought to not demonstrate substantial improvement over existing technologies, a key criteria for NTAP^7^. More recently, in June 2024 the American College of Radiology (ACR) submitted a letter to CMS recommending revisions to NTAP that would establish an alternative pathway for high-value AI technologies that would focus on three core facets: “newness” based on accomplishing a clinical task not previously possible; “uniqueness” based on addressing a clinical use case not addressed by existing technologies; and “value” based on both performance data and input from physician experts^16^.

Most recently, CMS announced a new coverage pathway, Transitional Coverage for Emerging Technologies (TCET), designed for technologies designated Breakthrough Devices by the FDA^17^. In their most recent notice, CMS indicated that they expect to accept up to five TCET candidates a year, with the goal of finalizing a national coverage determination within six months of FDA market authorization. The deadline for nominations for the first quarterly review is October 31, 2024, so we will soon see how this new pathway affects AI reimbursement.

Although the current healthcare environment primarily revolves around traditional fee-for-service reimbursement models, other authors have described additional reimbursement pathways. For example, Abramoff and colleagues describe a reimbursement framework that enables the analysis of value and cost for individual AI services^18^.

## How might reimbursement work for generalist radiology AI?

**Fig. 2:**
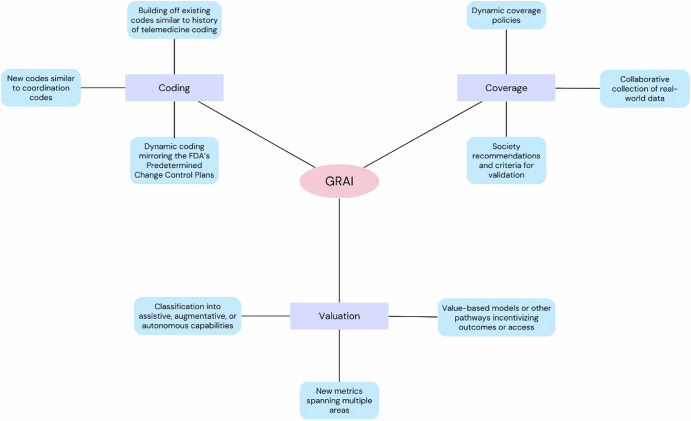
Reimbursement for generalist radiology AI. Three key areas of questions regarding reimbursement for generalist radiology AI (GRAI) along with possible approaches to help drive development of new pathways.

It remains to be seen how GRAI will be described and valued within the MPFS and other payment systems. Several questions will need to be answered, centering on three key areas: how will GRAI be coded, how will it be valued, and how will it be covered by other payors (Fig. 2)?

### Will GRAI receive its own CPT code?

Presently, there are two category I CPT codes and six codes with Category III status. Each of these is specific to a focused medical service, i.e., narrow AI. For instance, CPT 75580 specifically relates to FFR imaging within the coronary circulation. In contrast, GRAI is more general and functions across multiple diverse clinical activities and tasks.

One CPT code solution to describe GRAI may function more similarly to coordination codes within CPT rather than clinically specific radiology codes. CPT codes for care management describe coordinated activities which include and bridge existing clinical services. For example, CPT 99491 describes chronic care management services provided by a physician which includes the establishment and monitoring of a comprehensive care plan^19^. Such codes for GRAI may bridge different types of tasks depending on what it is utilized for (e.g., triage, diagnosis, report generation) as well as modalities and radiology subspecialties.

For example, a single multi-task coordination code could cover multiple tasks in a study, such as triaging a chest CT based on suspected pathology, detection and segmentation of abnormalities, and generation of structured reports. Such codes could be applied across multiple GRAI applications and types. Alternatively, a modality-specific code series could be developed with separate codes for the aforementioned levels of GRAI tasks which could allow for more granular differentiation, for example whether a brain MRI GRAI tool was used for only detection or for the full pipeline from triage to report generation. A code series may better align with the current reimbursement structure where complexity and modality impact valuation

More broadly, CPT coding for GRAI may parallel the history of telemedicine coding, which first reflected the idea of telemedicine as extensions of existing in-person care visits before development of telemedicine specific codes and modifiers^20^. GRAI coding could similarly start by building off existing radiology codes before it gets its own comprehensive framework. For example, new modifiers for brain MRI would indicate the level of GRAI involved, again ranging from detection/diagnosis to report generation. This strategy may scale more easily and avoid creating an overwhelming number of specific codes.

Another important consideration is that GRAI is inherently an evolving technology; by definition, it should be able to adapt to newly described tasks. Traditional CPT codes are designed for static technologies whereas GRAI will need a more adaptive strategy. The FDA faces similar challenges with AI/ML devices and has proposed Predetermined Change Control Plans (PCCPs) which will enable pre-approved modifications to these devices without needing full re-approval^21^. CMS should look closely at how the FDA implements PCCPs as similar strategies could help guide GRAI coding.

### If so, how will it be valued?

The RBRVS is a system based on relativity. As such new GRAI codes will be compared to other AI codes including those in radiology. Given the comprehensive nature of GRAI and the ability to apply it across multiple different tasks, will it be valued more highly on the RBRVS? For example, depending on the task and the specific needs of a given user, GRAI could be used in any or all of assistive (e.g., detects and highlights abnormalities in an imaging study for the radiologist), augmentative (e.g., long-term prognostication based on these abnormalities), or autonomous capabilities (e.g., generating full reports including differential diagnoses and recommendations).

As GRAI applications grow more autonomous, the complexity of its contributions increases and should be reflected in its valuation. Moreover, GRAI can be applied across numerous domains and so its valuation must rely on metrics that also span multiple areas leading to a composite, multifaceted RVU. For example, a composite GRAI RVU could account for radiologist work required for a given task, may include a higher task complexity component for autonomous tasks like report generation and an adaptability component for tools that integrate multi-modality data. It is likely that more AI/ML codes will undergo valuation and receive RVUs before GRAI, so these precedents will be relevant.

The ongoing adoption of AI tools may also spur changes in what we value and the underlying metrics that will affect GRAI’s valuation. Many authors have suggested alternative reimbursement pathways aside from free-for-service models, such as incentivizing outcomes or access^12,22^. Others have noted that value-based models may better address circumstances where the functions of AI tools can’t be appropriately divided into discrete services, which would certainly apply to many applications of GRAI^23^.

### What other steps will be necessary for coverage and diffusion into patient care?

Obtaining a CPT code and even RVU valuation does not guarantee adoption. Coverage decisions will be critical in determining if GRAI will be reimbursed by Medicare and other payors. Medicare coverage is influenced by national coverage determinations (NCDs) that apply to particular services or technologies across the entire United States as well as local coverage determinations (LCDs) that are set by Medicare Administrative Contractors (MACs) that process Medicare claims in specific regions. Meanwhile, private payors have their own criteria, which often but not always aligns with Medicare.

Although these criteria will vary, such as between payors or MACs, there are a number of universal elements under consideration such as demonstrated efficacy and safety of a tool, comparative effectiveness with respect to existing standards of care, cost effectiveness and utilization. All of these will necessitate collection of real-world data that studies GRAI in an array of clinical settings and applied to diverse patient populations. Indeed, there are already calls for more randomized trials of AI tools^24^. Collecting this data will likely be more difficult for GRAI compared to existing AI technologies given that it can be deployed in many more versatile situations; developers, clinicians, and payors must collaboratively plan how to gather necessary real-world data. Moreover, just as CPT codes are designed to work with static technology, coverage policies currently are not well equipped to deal with dynamic tools and must be revamped in anticipation of the emergence of GRAI. As society recommendations are also crucial for ensuring widespread coverage, the ACR and other radiology organizations should think about how best to validate GRAI.

The literature requirements to achieve coverage are similar to those necessary to earn a CPT code, but also could be different. For instance, greater focus on outcomes and technical considerations may apply.

## Conclusion

The advent of GRAI heralds a significant leap forward in the capabilities of radiology AI tools, promising to enhance clinical utility and flexibility. However, the successful integration of GRAI into widespread clinical practice hinges on developing a comprehensive and adaptive reimbursement framework. Unlike narrow AI, GRAI’s multifunctionality and ability to adapt to new tasks necessitate distinct reimbursement strategies. Payors and other stakeholders must proactively explore how GRAI payment would look in terms of coding, revenue, and coverage. Ultimately, both CMS and the AMA would need to establish an objective and transparent framework that incentivizes the adoption of high-quality, clinically impactful GRAI tools while also ensuring fair compensation for radiologists and healthcare providers.

## Data Availability

No datasets were generated or analysed during the current study.
